# Advances in Diagnosis and Therapy for Bladder Cancer

**DOI:** 10.3390/cancers14133181

**Published:** 2022-06-29

**Authors:** Xinzi Hu, Guangzhi Li, Song Wu

**Affiliations:** 1 Institute of Urology, The Affiliated Luohu Hospital of Shenzhen University, Shenzhen University, Shenzhen 518000, China; 2100243002@email.szu.edu.cn (X.H.); guangzhili@126.com (G.L.); 2 Department of Urology, South China Hospital, Health Science Center, Shenzhen University, Shenzhen 518116, China

**Keywords:** bladder cancer, diagnosis, non-invasive urine screening, intravesical or systemic therapy, liquid biopsy

## Abstract

**Simple Summary:**

The clinical management of bladder cancer has been developing in the past decade, including diagnostic tools and treatment options. Both monotherapy and combination therapy have been undoubtedly upgraded. Multiple diagnostic techniques and therapeutic strategies have been developed to meet the urgent clinical needs, resulting in the emergence of various explorations for cancer diagnosis and therapy. In this review, we mainly focus on the advances in the diagnosis and treatment of bladder cancer.

**Abstract:**

Bladder cancer (BCa) is one of the most common and expensive urinary system malignancies for its high recurrence and progression rate. In recent years, immense amounts of studies have been carried out to bring a more comprehensive cognition and numerous promising clinic approaches for BCa therapy. The development of innovative enhanced cystoscopy techniques (optical techniques, imaging systems) and tumor biomarkers-based non-invasive urine screening (DNA methylation-based urine test) would dramatically improve the accuracy of tumor detection, reducing the risk of recurrence and progression of BCa. Moreover, intravesical instillation and systemic therapeutic strategies (cocktail therapy, immunotherapy, vaccine therapy, targeted therapy) also provide plentiful measures to break the predicament of BCa. Several exploratory clinical studies, including novel surgical approaches, pharmaceutical compositions, and bladder preservation techniques, emerged continually, which are supposed to be promising candidates for BCa clinical treatment. Here, recent advances and prospects of diagnosis, intravesical or systemic treatment, and novel drug delivery systems for BCa therapy are reviewed in this paper.

## 1. Introduction

Bladder cancer (BCa) is one of the most common urinary system malignancies, with an estimated 80,000 new cases and 17,980 deaths worldwide in 2020 [1,2]. BCa can be mainly decided into non-muscle-invasive bladder cancer (NMIBC) and muscle-invasive bladder cancer (MIBC) according to the degree of tumor invasion. Among them, patients with NMIBC account for nearly 80% of cases initially diagnosed and are prone to suffer a risk of recurrence (~70%) and progression (~15%) after standard treatment of clinical guidelines. Approximately 25% patients are diagnosed with MIBC (T2a–T4b), the majority of them presenting with primary invasive BCa and a poor prognosis [3]. The high recurrence and progression rate of BCa aggravates the costly burden of patients for multiple tests and treatments. 

Urinary cytology and cystoscopy are the first-line approaches for the diagnosis of BCa. Cystoscopy is applied for the definitive diagnosis and surveillance of BCa, which also commonly suffers from infection and prostate injury in its invasive manner. Thus, the non-invasive testing technology with high specificity and sensitivity is urgently needed. Both the combination of optical techniques and novel imaging systems enhance diagnostic accuracy and reduce the risk. Moreover, urine-based non-invasive screening tests have become the hotspot of current research in recent years. Several urinary biomarkers have been developed for surveillance to avoid repetitious cystoscopy. Among them, six urine test markers (NMP22 BC, NMP22 BladderChek, BTA Stat, BTA TRAK, UroVysion, uCyt+/ImmunoCyt) have been applied for the clinical diagnosis of BCa and approved by the Food and Drug Administration (FDA). 

However, few of them are widely administrated in clinical practice for the limited specificity and equivocal clinical benefit [4,5]. To overcome such limitations, more urine-based non-invasive screening tests and tumor-associated biomarkers were discovered, including urine-derived protein, DNA methylation-based markers, and extracellular vesicles (EVs), promising approaches to decrease follow-up examination, providing additional feasibility in improving the diagnostic efficiency of BCa.

Intravesical instillation of chemotherapeutics or immunological pharmaceuticals after transurethral resection of bladder tumor (TURBT) is commonly performed as adjuvant therapy for NMIBC [6], which has been proved to be an effective way to eliminate the residual tumor cells after operation to avoid recurrence [7]. Strategies for MIBC include neoadjuvant therapy, radiotherapy, radical cystectomy (RC), or partial cystectomy [2]. Although the above clinical interventions could partly alleviate the tumor recurrence and progression, a large proportion of patients deteriorate into high-grade or metastatic disease, suffering from cisplatin-based cytotoxic chemotherapy with a poor prognosis (5-year progression rates range from 0.8% to 45%) [2,8]. Novel therapies, including tumor-targeted drugs, antibody conjugated drugs, immune checkpoint inhibitors, and vaccines, have been developed to meet the treatment needs of patients with BCG failures. In addition, abundant novel drug delivery carriers have been designed to improve the efficacy of instillation therapy. For patients with advanced BCa, improved neoadjuvant therapy and novel therapeutic modalities play an active role in clinical management [2]. The aim of this review is to provide a comprehensive summary of the advances in BCa diagnosis and treatment, which will introduce different therapeutic approaches for BCa.

## 2. Diagnosis

### 2.1. Optical Techniques and New Imaging Systems

With standard white-light cystoscopy (WLC), it is easy to miss the minimal residual tumor tissues during the diagnostic detection and resection of carcinoma in situ (CIS) [9]. To overcome this dilemma, diagnostic technology is constantly being upgraded, where laser-induced fluorescence (LIF), autofluorescence cystoscopy (AFC), and photodynamic diagnosis (PDD) are becoming the focus of attention.

#### 2.1.1. Photodynamic Diagnosis

**Figure 1:**
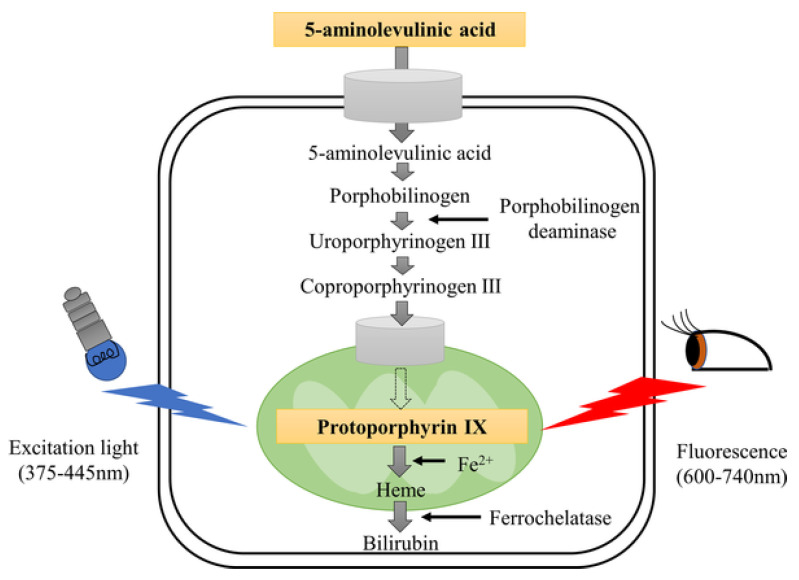
Schematic illustration of the mechanism of PDD. Reproduced with permission from Ref. [13], Copyright (2021) Sasaki et al.

PDD involves the instillation of a photosensitizer (5-ALA: 5-aminolaevulinic acid; HAL: hexaminolevulinate) into the bladder before cystoscopy. Tumor cells absorb the photosensitizers and show red fluorescence under blue light (380–450 nm) exposure based on the different enzymatic activity between malignant and benign tissues, which is helpful in distinguishing tumor cells from para-cancer tissues [10] (Figure 1). A previous meta-analysis from Xiong et al. noted the tumor recurrence rate in the 5-ALA- based PDD group is significantly lower than that in the HAL-PDD group (Odds ratio [OR]: 0.48, 95% CI [confidence interval]: 0.26–0.95) [11,12]. Burger et al. reported that the detection rate of CIS lesions by PDD was 40.8%, which is higher than that by WLC [9]. According to a meta-analysis by Russo et al., PDD also showed a higher diagnostic OR and sensitivity than WLC [10]. 

TURBT combined with PDD can maximize the tumor resection rate to reduce the recurrence of BCa. The prospective evaluation study of PDD for NMIBC surveillance noted that 33% of additional cancers were detected by PDD, and the recurrence rate of NMIBC detected and resected by PDD was lower than that by WLC [14]. This result is consistent with the findings of the recurrence-focused randomized study from Drejeret al. The use of cystoscopy-based PDD for NMIBC surveillance after the first-time TURBT reduced the risk of recurrence with an OR of 0.67 (*p* = 0.02, 95% CI: 0.48–0.95) [15]. According to a systematic review by Veeratterapillay et al., 2288 patients from 12 randomized controlled trials were included in the meta-analysis, which found that PDD reduced recurrence rate and improved recurrence-free survival (RFS) (68.2% vs. 57.3%) for NMIBC over at least a 2-year follow-up period compared with WLC [16]. Motlagh et al. noted that PDD combined with immediate intravesical chemotherapy resulted in an additional 32% reduction OR for the 12-month recurrence risk [17]. 

#### 2.1.2. Fluorescence Cystoscopy

Similar to PDD, LIF is involved in light emission and excitation, as well as the absorption of endogenous porphyrin compounds. The difference in spectra is mainly the result of cellular oxygenation processes and reduction between tumor cells and normal tissues, as evidenced by changes in the NAD to NADH ratio [18]. For the past years, AFC was commonly applied as a complementary tool to standard cystoscopy. Optical filters and algorithms convert images into spatial maps of the intensity of autofluorescence, which can significantly improve sampling accuracy of tissue biopsies and pathology examinations. The Onco-LIFE system (photo-induced fluorescence endoscopy) provides an objective comparison of green and red self-fluorescence by calculating a ratio (NCV: numerical color value), which enables precise localization of small pathological changes [18,19]. Compared to WLC, AFC and PDD exhibit higher sensitivity and better ability to perform biopsies. 

#### 2.1.3. Optical Biopsy Techniques

Some optical biopsy techniques, including optical coherence tomography (OCT) and confocal laser endomicroscopy (CLE), involve specific wavelengths of light and cystoscopy in dynamic real-time images of tissue for the surveillance of BCa [19,20]. Sonn et al. suggested that CLE is an effective aid to cystoscopy using dye fluorescein and light from a 488 nm laser fiber optic source to provide real-time dynamic images of malignant cells and normal tissues [21]. 

Similarly, OCT utilizes near-infrared light (890–1300 nm) to scatter tissue layers, providing tissue images with a penetration depth of 2 mm and a spatial resolution of 10–20 μm. The measurement of light scattering is performed by comparing a back-scattered or back-reflected light signal to a reference signal, which is highly sensitive and specific to identify malignant lesions [22]. According to a meta-analysis by Brunckhorst et al., OCT could remarkably improve overall diagnostic accuracy with a specificity of 60–98.5% and a sensitivity of 74.5–100% [20]. A meta-analysis of OCT for BCa identification by Xiong et al. noted that the sensitivity, specificity, and the area under the receiver operating characteristic (AUC) were 94.9% (95% CI: 92.7–96.6%), 84.6% (95% CI: 82.6–86.4%), and 0.97, respectively [23]. 

In addition, some novel spectral and imaging techniques have been introduced into diagnosing BCa, including diffuse reflectance spectroscopy, Raman spectroscopy, elastic light scattering, vibrational spectroscopy, biophotonic methods, multi-photon microscopy, and scanning fiber endoscopy [19,20]. Overall, combinations of imaging modalities offer higher quality benefits than diagnostic methods alone, such as PDD and NBI, PDD and CLE, as well as CLE and OCT, have been considered. Schmidbauer et al. evaluated 66 patients with suspected BCa by using WLC, PDD, and PDD combined with OCT. The result showed an increase in sensitivity from 89.7% to 100% and specificity from 62% to 87% [22]. 

#### 2.1.4. Imaging

Similar to PDD, NBI (narrow-band imaging) provides a three-dimensional image of the bladder to distinguish between intensive vascular tumors and normal tissues. NBI is a novel cystoscopy-aid imaging strategy [9,10]. Kutwin et al. reported that the sensitivity of NBI for BCa detection was 94–97.9%, compared with WLC (87–88.8%). Moreover, the sensitivity of NBI for CIS was remarkably superior to WLC (93–100% vs. 66.7–77%) [9]. In a recent meta-analysis, the additional detection rate of NBI for NMIBC showed 18.6% greater than that of WLC [10]. However, no significant difference between PDD and NBI in sensitivity and specificity was found in current studies. Both effective methods increase the visibility of cystoscopy and can prolong the follow-up interval of recurrence or progression [10,24,25]. 

As auxiliary strategies, cross-sectional urography, including computed tomography (CT) and magnetic resonance imaging (MRI), is commonly used to detect large masses or invasive tumors in the upper urinary tract [26,27]. Diffusion-weighted imaging (DWI) and apparent diffusion coefficient (ADC) provide valuable methods for distinguishing between peripheral tissue and tumor invasion [19]. DWI is a non-invasive functional imaging method that has been widely used for histological grading and radio sensitivity examination in malignant tumors [28,29]. The DWI signal is assessed by visual image interpretation and the quantitative analysis of ADC, and local staging is performed based on the difference in signal intensity [28,29]. Texture analysis of ADC maps and texture features selection predict chemoradiotherapy response and identify the classification of pathological tumor response in MIBC [29]. DWI plays a potential role as a functional magnetic resonance imaging technique for the qualitative and quantitative detection of BCa. Yoshida et al. reported that the sensitivity, specificity, and accuracy of DWI for the diagnosis of BCa were 91–100%, 77–91%, and 81–96%, respectively [28]. However, inflammatory and granulomas may appear high-intensity DWI signals as well, leading to the risk of false-positives [28]. Moreover, Cai et al. reported that the overall diagnosis of histological grading for BCa with synthetic MRI-derived parameters was inferior to ADC. Still, the efficiency of the former was much better than that of ADC due to multiple contrast-weighted images and quantification maps generated in a single scan [30]. 

There is also a potential role for emerging computer-based systems in BCa diagnosis. The CAD system, a multi-parametric computer-aided diagnosis system based on magnetic resonance T2W imaging and DWI, is applied for diagnostic differentiation of BCa staging, especially T1 and T2 stages. Hammouda et al. reported that the total area under accuracy, sensitivity, and specificity of CAD system were 95.24%, 95.24%, and 95.24%, respectively [31]. 

VI-RADS (Vesical Imaging-Reporting and Data System), a novel multi-parametric system, was developed to standardize the reporting and staging of preoperative multi-parametric magnetic resonance imaging of BCa, which involves T2W imaging, dynamic contrast-enhanced MRI, and DWI [2,32,33,34]. The VI-RADS scores are based on the signal intensity of T2W imaging, DWI, ADC, and dynamic contrast enhancement (DCE) MRI in different layers of the bladder wall. According to a retrospective study by Meng et al., the overall AUC value of VI-RADS was 0.939 with a cutoff value of 3 or greater [33]. In a meta-analysis included six studies with more than 1000 patients, the sensitivity and specificity of detection for MIBC by VI-RADS were 0.90 (95% CI: 0.86–0.94) and 0.86 (95% CI: 0.71–0.94), respectively [35]. Moreover, Ahn et al. noted that tumor contact length could be used as a complementary indicator for VI-RADS to predict MIBC at a threshold of 3 cm to reduce the false-positive rate [36]. A single-center retrospective study showed that the specificity of the integration of VI-RADS and tumor contact length was 82.46–87.72%, and the PPV was 90.91–91.59%, indicating an effective strategy to reduce the false-positive rate of VI-RADS [37]. Feng et al. proved that the integration of fractional-order calculus model and VI-RADS increased the AUC value from 0.859 to 0.931, which helped to identify and stage BCa [38]. Thus, VI-RADS may be the most useful method in accelerating radical treatment and determining response to bladder preservation methods for NMIBC [34].

#### 2.1.5. Ultrasound

Ultrasound is an effective method for BCa detection. The 29 mhz high-resolution micro-ultrasound (mUS) technique has been suggested as an alternative method for detecting BCa and differentiating between NMIBC and MIBC, which provides real-time images and a cost effectiveness approach. A comparison study of the diagnostic accuracy of mUS vs. MRI in distinguished NMIBC and MIBC at definitive pathological examination. The sensitivity, specificity of mUS and MRI were 85.0% vs. 76.3% and 85.0% vs. 50.0%, respectively [39]. 

Contrast-enhanced ultrasonography (CEUS) is another novel ultrasound techniques, which is used for the differentiation of high- and low-grade urothelial carcinoma. A sensitivity of 86% and specificity of 90% were obtained for high-grade tumors, while a sensitivity of 85% and specificity of 89% were obtained for low-grade BCa [40]. Moreover, Li et al. introduced a combination diagnosis of CEUS and MRI+DWI, and the accuracy of the combination diagnosis was higher than that of the single diagnostic methods [41].

#### 2.1.6. Novel Diagnostic Systems

**Figure 2:**
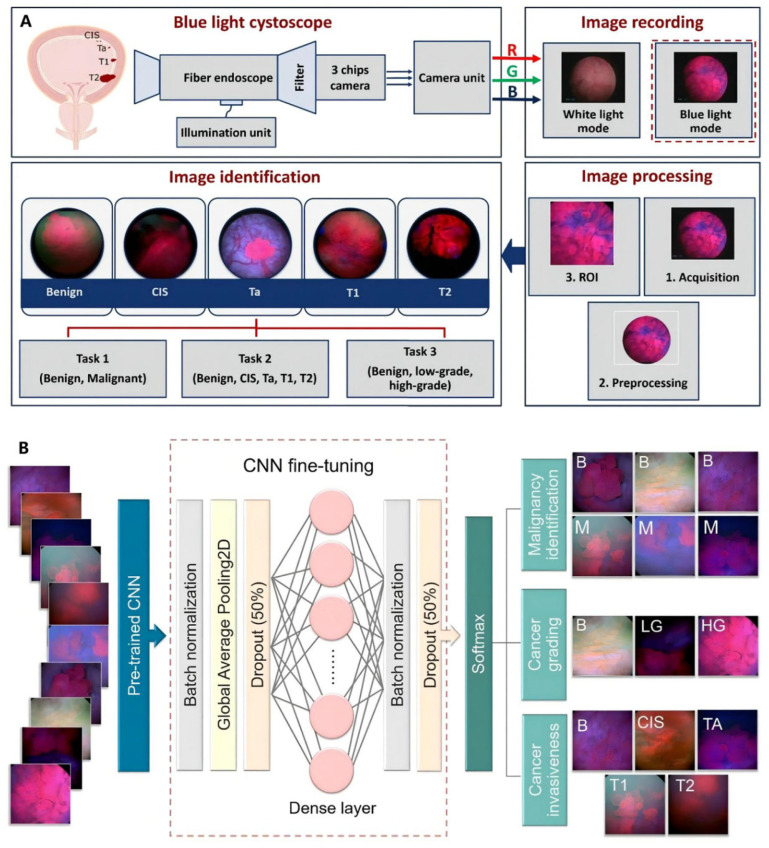
AI model based on blue light cystoscopy for BCa diagnosis. (**A**) Overview of image acquisition and image processing using blue light cystoscopy. (**B**) Schematic diagram of the CNN fine-tuning considered for identifying BCa. Reproduced with permission from Ref. [43], Copyright (2021) Nairveen Ali et al.

In recent years, studies have involved artificial intelligence (AI) models in diagnostic methods for BCa. Deep learning is a new area of AI, which has been introduced in BCa management, including automated tumor detection, staging and grading, bladder wall segmentation, and tasks such as recurrence prediction, chemotherapy response, and overall survival evaluation. Shkolyar et al. developed a convolutional neural network-based image analysis platform (CystoNet) for the automatic detection of BCa with a sensitivity of 90.9% (95% CI: 90.3–91.6%) and a specificity of 98.6% (95% CI: 98.5–98.8%) [42]. Ali et al. introduced an AI diagnostic platform, which depended on four pre-trained convolutional neural networks (CNN) to predict the malignancy, invasiveness, and grading of the images with a sensitivity of 95.77% and a specificity of 87.84%, respectively [43]. The classification pipeline for AI detection of malignant tumor task and the detailed process of it is illustrated in Figure 2. However, the over-diagnosis of AI detection is reported to be concerned. Thus, there is a need to improve recognition and tasks of AI detection, including tumor staging and grading [44].

At present, many challenges may be faced before these new technologies become mainstream for the equipment requirement. These complementary diagnostic methods bring higher quality for BCa diagnosis, and the perfect optical techniques and imaging systems gradually replace histopathological analysis. To assess the actual value, more random studies are needed to determine the potential of these techniques in BCa diagnosis.

### 2.2. Urine Tests and Biomarkers

**Figure 3:**
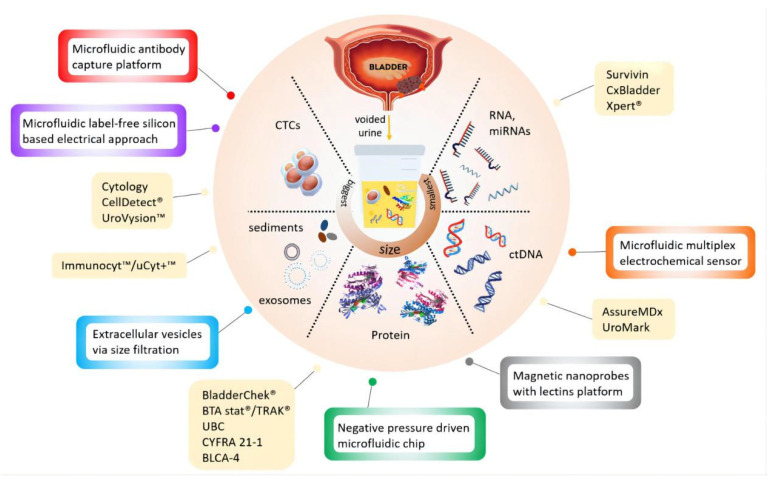
Current available (yellow boxes) and potential devices (color bordered boxes) for urinary BCa diagnosis. Reproduced with permission from Ref. [49], Copyright (2020) Kit Man Chan et al.

Urine cytology is a commonly used non-invasive test for the clinical management of BCa, which exhibits good sensitivity for CIS and high-grade BCa, while showing poor performance for low-grade tumors. Urine-based non-invasive screening tests have demonstrated superior potential clinical effectiveness, and many biomarkers involving proteins, DNA methylation, and EVs have been discovered. The current urine-based biomarkers and assays refer to Figure 3. Six urine biomarkers approved by FDA have been applied for the diagnosis and monitoring of BCa, including NMP22 BC (nuclear matrix protein 22 ELISA test), NMP22 BladderChek, BTA Stat (qualitative test), BTA TRAK (quantitative test), UroVysion (FISH), and uCyt+/ImmunoCyt (fluorescent immunohistochemistry) (Table 1) [45,46]. The sensitivity of most tests increases with tumor stages or grade, but false positives can occur due to the possibility of inflammation and hematuria. Although their sensitivity is superior to urine cytology, they still have not replaced the current diagnostic criteria of the test [47,48]. Therefore, we still need more effective detection methods to detect early and minimal tumors.

#### 2.2.1. Proteins

Some protein-based biomarkers and assays that have not yet been clinically recommended have also progressed besides NMP22 BC and BTA assays. CYFRA21-1 is a cytokeratin 19 fragment and is reported to be a promising biomarker for diagnosing or monitoring the prognosis of BCa [50]. Matuszczak et al. noted that CYFRA21-1 is highly sensitive for diagnosing CIS and high-grade BCa [51]. According to a meta-analysis by Huang et al., an ELISA test for CYFRA21-1 that detects the soluble fragments of cytokeratin 19 in urine showed the sensitivity and specificity were 82% (95% CI: 0.70–0.90) and 87% (95% CI: 0.84–0.90), respectively [52]. Lei et al. invented a fluorescent nanosphere-based immunochromatographic test strip for CYFRA21-1 with a sensitivity of 92.86% and a specificity of 100% for BCa diagnosis [53].

Urinary Bladder Cancer (UBC) ELISA and UBC immunoradiometry are used to detect the level of cytokeratin 8 and 18 fragments in urine. The results of a meta-analysis by Lu et al. showed the sensitivity and specificity were 59% (95% CI: 55–62%) and 76% (95% CI: 72–80%), respectively [54]. Meisl et al. developed a nomogram based on a multi-center dataset to identify patients with high-risk BCa, and urine was analyzed using the UBC^®^ Rapid test. The results showed that the risk factor-based nomogram had a predictive area of 0.79 (95% CI: 0.72–0.87) and 0.95 (95% CI: 0.92–0.98) for low-grade Bca and high-grade Bca, respectively, which can be a helpful screening tool for NMIBC [55].

Survivin, a member of the apoptosis suppressor gene family, is associated with cell apoptosis, proliferation, cell cycle, angiogenesis, and tumor cell survival. Liang et al. noted that the total sensitivity and specificity of survivin were, respectively, 79% (95% CI: 0.73–0.84) and 87% (95% CI: 0.79–0.92) [56].

BLCA-1 [57] and BLCA-4 [47], two transcription factors of the nuclear matrix protein, have promising prospects in the diagnosis of early tumors with sensitivity and specificity, respectively, 80% and 93% (95% CI: 0.90–0.95) and 87% and 97% (95% CI: 0.95–0.98).

Microchromosome maintenance protein 5 (MCM5) is a crucial factor in DNA replication, located at the basal layer of the epithelium in normal tissues, which would extend to the whole epithelial layer in the tumor situation. A commercial ELISA kit, named ADXBLADDER, was applied for the urine-based non-invasive screening for BCa, depending on the detection of MCM5 level with an overall sensitivity of 44.9% (95% CI: 36.1–54) and specificity of 71.1% (95% CI: 68.5–73.5) [58]. A multi-center study by Roupret et al. demonstrated a negative predictive value (NPV) of 99.15% for ADXBLADDER to preclude high-grade/CIS recurrence [59].

URO17 assay is a urine test to detect the level of keratin 17 (K17) in BCa patients with high sensitivity. According to Babu et al. study, the expression of K17 in 112 urine was applied for the BCa diagnosis via immunocytochemistry analysis, with a sensitivity and specificity of 100% and 96%, respectively [60]. These urine-based assays are more sensitive than urine cytology, but they tend to be less specific and sensitive than WLC for low-grade BCa (30–60%).

Oncuria™, a multiplex immunoassay, was used to detect bladder performance in urine in a multi-institutional cohort study. For a total of 362 prospectively collected subjects evaluated for BCa, Oncuria™ had an overall sensitivity of 93%, specificity of 93%, PPV of 65%, and NPV of 99% [61].

#### 2.2.2. Genomic Biomarkers

Genomic biomarkers have also shown the effectiveness in the diagnosis of BCa. Microsatellite analysis utilizes PCR to analyze DNA mutations in urinary exfoliated cells with an overall sensitivity of 58–92% and a specificity of 73–100% [62]. Telomeric repeat amplification (TRAP) is used to detect telomerase with high sensitivity (90%) and specificity (88%) [63].

Fibroblast growth factor receptor 3 (FGFR3) presents in 70% of NMIBC, and Zuiverloon et al. reported the sensitivity of FGFR3 for detecting recurrence of BCa was 58% [64]. The Quanticyt system could automatically quantitative cell nucleus with a sensitivity of 59% and a specificity of 79%, while little advanced research has been reported in the past decades [65].

According to a study by Lokeshiwar et al., hyaluronic acid- hyaluronidase (HA-HAase) has a sensitivity of 91% and a specificity of 70% for detecting BCa. Still, the risk of recurrence within five months is high in those with false positives [66]. Over-expression of eukaryotic initiation factor 5A2 (EIF5A2) and the AIB1 gene is associated with postoperative recurrence of BCa [67]. Chen et al. developed a combined EIF5A2, AIB1, and NMP22 assay model with a sensitivity and specificity of 92%, which is superior to single biomarker assays [68].

Cxbladder detects four mRNAs (IGFBP5, HOHA13, MDK, CDK1) in urine to diagnose BCa and monitor recurrence. The pooled sensitivity, specificity, positive predictive value (PPV), and NPV were 91% (95% CI: 0.85–0.95), 61% (95% CI: 0.21–0.90), 16% (95% CI: 0.09–0.28), and 98% (95% CI: 0.82–0.99), respectively, as reported in Laukhtina’s meta-analysis [69].

The XPERT© Bladder Cancer Monitor is a test for detecting the five mRNA sequences (ABL1, CRH, IGF2, UPK1B, ANXA10) in urine [70,71]. The sensitivity, specificity, PPV, and NPV of another urinary biomarker test for XPERT© Bladder Cancer Monitor were 72% (95% CI: 0.63–0.80), 76% (95% CI: 0.72–0.81), 43% (95% CI: 0.32–0.54), and 92% (95% CI: 0.90–0.90), respectively [69]. A prospective study from Singer et al. noted that the XPERT© Bladder Cancer Monitor might provide better sensitivity in the case of high-grade NMIBC recurrence [61].

Moreover, Uromonitor is an effective urinary biomarker test for monitoring BCa recurrence, and its sensitivity, specificity, PPV, and NPV were, respectively, 93% (95% CI: 79–98%), 79% (95% CI: 62–90%), 67% (95% CI: 36–89%), and 96% (95% CI: 86–99%). Uromonitor and Cxbladder are not feasible to monitor the recurrence of high-grade BCa due to the lack of data [69] (Table 2).

#### 2.2.3. DNA Methylation

**Figure 4:**
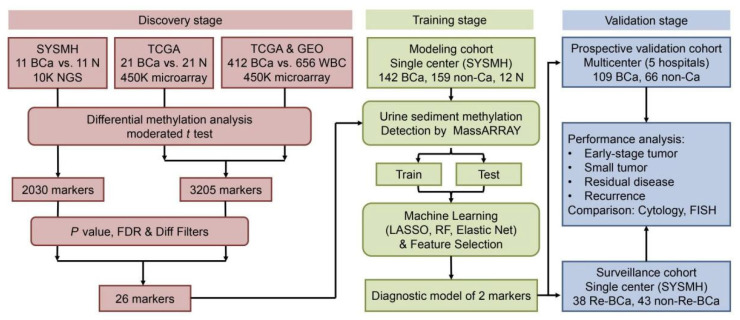
Study design and workflow of utMeMA. (SYSMH = Sun Yat-sen Memorial Hospital; TCGA = the Cancer Genome Atlas; BCa = bladder cancer; FDR = false discovery rate; LASSO = the least absolute shrinkage and selection operator; RF = random forest). Reproduced with permission from Ref. [72], Copyright (2020) American Society for Clinical Investigation.

Metabolites can be applied as urinary biomarkers. Several DNA methylation biomarkers are one of the leading research topics. Interestingly, utMeMA, a DNA methylation-based assay for detecting multiple genomic regions of urinary tumors, was reported by Lin and colleagues [72] (Figure 4). DNA methylation markers come from a combined analysis of three cohorts from Sun Yat-sen Memorial Hospital (SYSMH), the Gene Expression Omnibus database (GEO), and The Cancer Genome Atlas (TCGA). The integrated analysis of BCa sequencing data from three cohorts identified 26 BCa-specific methylation sites with sensitivity and specificity of 90% and 83.1%, respectively. The utMeMA-based assays have greatly improved the detection sensitivity for early BCa (Ta stage and low-grade BCa) [72].

Several DNA methylation-based assays have also been reported. Bladder EpiCheck is a DNA methylation profile-based assay that analyzes DNA in spontaneous urine, with a sensitivity, specificity, PPV, and NPV of 74% (95% CI: 57–85%), 84% (95% CI: 80–88%), 48% (95% CI: 42–54%), and 94% (95% CI: 90–97%), respectively [69,73]. Several clinical trials have documented that the Bladder EpiCheck methylation test is an effective method for surveillance of high-risk NMIBC [74,75].

UroMark assay, a bisulphite sequencing assay and analysis pipeline for detecting BCa from urinary sediment DNA with a sensitivity, specificity, and NPV of 98%, 97%, and 97%, respectively [76].

The Bladder CARE test uses methylation-sensitive restriction endonucleases to measure the methylation levels of three BCa-specific biomarkers, which had an overall sensitivity, specificity, PPV, and NPV of 93.5%, 92.6%, 87.8%, and 96.2%, respectively, potentially improving the detection of early BCa [77].

The GynTect^®^ assay, a method based on six DNA methylation-based markers, was initially designed to diagnose cervical cancer and was applied by Steinbach et al. to detect BCa with a sensitivity and specificity of up to 60% and 96.7% [78]. (Table 3).

#### 2.2.4. Extracellular Vesicles

EVs contribute to the development and progression of BCa by influencing the cell cycle, facilitating the epithelial to mesenchymal transition, and forming the tumor mesenchyme. EV-derived macromolecules act at different stages of BCa tumorigenesis [79]. In recent years, many EVs have been discovered and show potential promise for BCa detection. Some genetic substances and EVs, shown in Table 4, have been proved as potential biomarkers. The technical approach for capturing and isolating EVs by double nanofiltration has been developed, with a sensitivity of 81% and a specificity of 90% [80]. Furthermore, several urinary biomarker-based microdevices have been reported, which used microdevice-assisted methods providing real-time detection needed only microliters of urine with the specificity and sensitivity for cancer cells being over 95% [49] (Table 4). In addition, Miyake et al. created a device named cellular fluorescence analysis unit-II (CFAU-II), which introduced cellular fluorescence analysis into urine cytology with an overall sensitivity of 63% (*p* < 0.001) [81].

Overall, the non-invasive and highly specific biomarkers in urine have shown great promise in the diagnosis and surveillance of BCa. However, the effectiveness of urine biomarkers for BCa diagnosis is not accurate enough, with ambiguous clinical benefits for early low-grade BCa. Moreover, the concentrations of biomarkers may vary with the organ function and medications. For the facility, parts of biomarkers are needed special techniques, which call for the requirement of highly qualified personnel and comprehensive equipment [84]. In short, the evaluation of the performance of biomarkers requires large prospective multi-center studies for the replacement of cytology.

## 3. Intravesical Therapy

### 3.1. Early Instillations and First-Line Therapies

Intravesical pharmaceutics or immune agents is widely used as the first-line treatment for NMIBC [2]. Mitomycin C (MMC) and bacillus calmette-guérin (BCG) are common agents for NMIBC perioperative treatment. However, the clinical application of MMC is limited by toxicity and cumulative myelosuppression after systematic administration [2,85]. The response rate of BCG induction was 50% for patients with CIS, resulting in most patients receiving additional maintenance therapy [86].

### 3.2. Therapies in Unresponsive BCG

**Figure 5:**
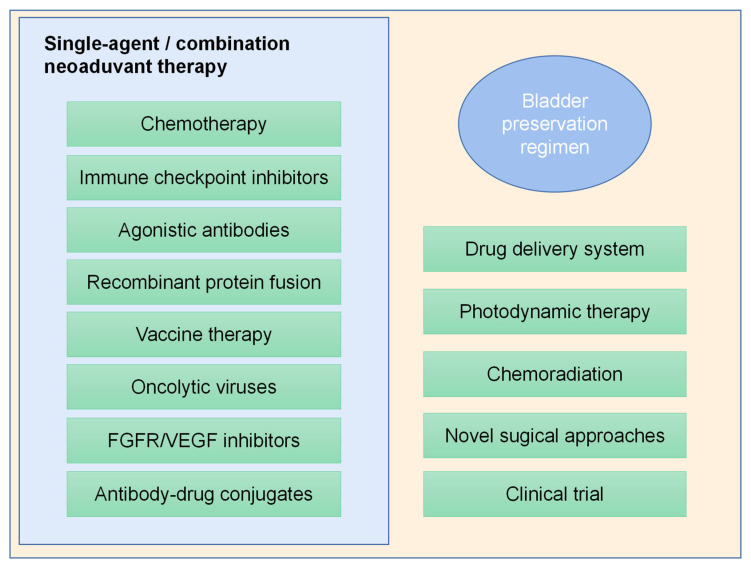
Novel treatment options as bladder preservation regimens for BCa.

Agents, including doxorubicin, epirubicin, and valrubicin, are widely employed for intravesical instillation as alternative options to BCG [87]. Figure 5 summarizes novel treatment options as bladder preservation regimens for patients with unresponsive BCG. 

In a randomized clinical trial (SWOG S0337), 406 patients with suspected low-grade NMIBC underwent intravesical instillation immediately after TURBT. In total, 34% of them in the gemcitabine group recurred within four years, which was superior to the 4-year recurrence rate of 54% in the saline group (HR: [hazard ratio]: 0.53; 95% CI: 0.35–0.81) [88]. Di Lorenzo et al. compared the efficiency between gemcitabine and BCG, and they showed that patients with high-risk NMIBC who received intravesical gemcitabine had significantly better recurrence than BCG alone (52.3% vs. 87.5%) [89]. In a sequential salvage gemcitabine and MMC combination study, 27 patients with BCG failures were included. In this group, 2-year disease-free survival (DFS) was 37%~38%, and the progression rate was 19% after receiving chemotherapy. The regimen was generally well tolerated, with most adverse events associated with nausea caused by gemcitabine or MMC components [90,91]. Moreover, intravesical gemcitabine could be used as a bladder preservation therapy for NMIBC with BCG failure, which has proven to be an alternative option to BCG with a DFS of 32.69% at 36 months [92].

Cabazitaxel is a paclitaxel-type chemotherapeutic agent commonly used to treat prostate cancer [93]. In a phase 2 study (SECAVIN) involving 70 patients with advanced localized or metastatic urothelium transitional cell carcinoma, three patients (13%, 95% CI: 2.7–32.4) in the cabazitaxel group achieved partial remission, while at the same time, six patients (30%, 95% CI: 11.9–54.3) in the vinflunine group achieved partial remission. Median progression-free survival (PFS) was 1.9 months vs. 2.9 months (*p* = 0.039). The study was not conducted in the phase 3 trial due to the lack of efficacy of cabazitaxel, as demonstrated by the futility analysis [94]. However, intravesical therapy with gemcitabine, cabazitaxel, and cisplatin (GCP) was well-tolerated, and a complete response rate (CRR) of 78% was obtained. This is a promising treatment option for BCG-unresponsive patients, with a 94% CRR and 78% DFS at 9.5 months [90,91]. In a phase 1 study by DeCastro et al., BCG-naive patients were treated with GCP showed a 1-year relapse-free survival (RFS) rate of 83% (95% CI: 0.57–0.94) and a 2-year estimated RFS rate of 64% (95% CI: 0.32–0.84) [95].

Docetaxel acts by blocking microtubule depolymerization, leading to cell cycle arrest and cell death [96]. In the phase 2 study by Kim et al., patients with advanced or metastatic BCa who had progressed after platinum-based chemotherapy received docetaxel chemotherapy. Two patients (6%) maintained an objective response for 3.0 to 7.8 months. Eight patients had stable disease with a disease control rate of 32%. Median PFS and overall survival (OS) were, respectively, 1.4 months (95% CI: 1.3–1.6 months) and 8.3 months (95% CI: 5.9–10.6 months). Fatigue is the most common adverse reaction [97].

Apaziquone is a benzoquinone-based bioreductive drug initially designed for the intravesical treatment of NMIBC [98]. Karsh et al. noted that instillation with apaziquone within 60 ± 30 min after TURBT would achieve a 20.3% reduction in 2-year recurrence rate and a 56% reduction in recurrence. Although the results were not statistically different from the placebo instillation group of that, the safety profile was excellent [99]. However, no new trials being recruited, and the development of apaziquone appears to be stagnant.

The efficacy of combination therapy is much better than a single drug alone, which has been proved in treating unresponsive BCG or BCG-ineligible patients [100]. A study reported that intravesical BCG regimen with epirubicin and IFN used for NMIBC, the BCG group had a significantly lower incidence of recurrence than that in the epirubicin/IFN group. The median follow-up time (7.4 years) was 39% and 72%, respectively (HR: 0.41; 95% CI: 0.28–0.60). There were no significant differences in the probability of progression or OS. In addition, gemcitabine and docetaxel (Gem/Doce) are one of the most potential intravesical salvage regimens, with a DFS of 42–54% and 27–37% at 1 and 2 years, respectively, which was well-tolerated by patients [90,101,102]. Steinberg et al. compared the efficacy of BCG/IFN and Gem/Doce with each other for patients with relapsed NMIBC after a single course of treatment. The 1-year and 2-year RFS were 61% and 53% for BCG/IFN, and 68% and 46% for Gem/Doce, respectively, and there was no a significant difference in results [103]. The efficacy of Gem/Doce was evaluated in two retrospective studies. DFS at 1 and 2 years were 42–54% and 27–37%, respectively. However, about 10% of patients experienced disease progression to MIBC [90,104].

## 4. Immunotherapy

### 4.1. Immune Checkpoint Inhibitors

The first-line treatment for MIBC patients is cisplatin-based chemotherapy. Patients with MIBC who are refractory to chemotherapy call for optimized cisplatin-based neoadjuvant chemotherapy, and the potential option is immune checkpoint inhibitors (ICIs) [105]. Currently approved ICIs are targeting cytotoxic T lymphocyte-associated protein 4 (CTLA4), programmed cell death-1 (PD-1), and its ligand (PD-L1) [106]. PD-1 and PD-L1 play a key role in T cell co-inhibition and exhaustion. Interaction of PD-1 and PL-L1 inhibits T-cell function and allows tumor cells to evade the immune response. However, the over-expression of them on tumor cells and lymphocytes is associated with poor prognosis in some human cancers [107]. The primary mechanism involved tumor cells (IDO) evading immune control and recruiting immunosuppressive cells (Treg cells and myeloid-derived suppressor cells (MDSCs)) to inhibit T cells by producing immunosuppressive transforming growth factor-β(TGF-β) and indoleamine 2, 3-dioxygenase. Monoclonal antibodies that lead to the blockage of the PD-1/PD-L1 pathway have been developed for immunotherapy of BCa via improving T cells function [108,109]. However, biomarkers for predictive response to ICIs remain an unmet need in the management of metastatic diseases. The study’s results demonstrated a survival benefit in PD-l1-positive mUC patients treated with ICIs, which could serve as a valid predictive biomarker for ICIs therapy [110]. A comprehensive table summarizes results of immunotherapies and targeted therapies reported in this review (Table 5).

#### 4.1.1. Anti-PD-1/PD-L1 ICIs

Five ICIs, including anti-PD-1 (pembrolizumab, nivolumab) and anti-PD-L1 (atezolizumab, durvalumab, avelumab) antibodies targeted for the PD-1/PD-L1 pathway, have been approved by the FDA for the treatment of BCa. In recent years, pembrolizumab (anti-PD-1) was approved by the FDA for patients with BCG unresponsive CIS and who are unwilling or ineligible for RC [86,111,112]. In phase 3 (KEYNOTE-045) study by Vaughn et al., 519 patients were included in the health-related quality-of-life analysis (pembrolizumab, *n* = 266; chemotherapy, *n* = 253). Patients who were treated with pembrolizumab experienced increasing OS (10.3 vs. 7.4 months; HR: 0.73; 95% CI: 0.59–0.91) at the median follow-up of 14.1 months (range 9.9–22.1) [113]. Another phase 2 study (KEYNOTE-057) by Balar et al. showed that 39 (41%) of 96 patients with BCG-unresponsive CIS who received pembrolizumab had an early durable CRR of 40% within 3 months, and most patients achieved a CRR of >6 months. Grade 3 or 4 treatment relative adverse events appeared in 13% of patients, and the most adverse events were arthralgia (in 2% of patients) and hyponatremia (in 3% of patients) [114,115].

Biomarkers can predict the prognosis and efficacy of immunotherapy. Evidence proved that comprehensive tumor mutational burden, genomic profiling (somatic mutation in the TP53, EZH2, APC, TERT, CDKN1A, CDKN1B, and ARID1A genes, and truncation in the BRCA2 gene), and PD-L1 expression (combined positive score) might serve as biomarkers for predicting pathological complete response of MIBC treated with neoadjuvant pembrolizumab [100,116]. The phase 2 trial study (PURE-01) by Necchi et al. indicated that pembrolizumab could be effective neoadjuvant immunotherapy for the treatment of MIBC with PD-L1-positive or high TMB tumors [117]. New findings suggested that patients with squamous cell carcinoma or lymphoepithelioma-like features might be suitable for pembrolizumab neoadjuvant immunotherapy [118,119]. It is worth noting that antibiotic therapy may have a detrimental effect on the efficacy of neoadjuvant pembrolizumab [120].

Atezolizumab is an FDA-approved anti-PD-L1-antibody as a second-line treatment for advanced BCa, and was well tolerated in most studies for BCa [121]. A long-term phase 1 study by Petrylak et al. reported that 95 patients with metastatic disease receiving atezolizumab (MPDL3280A) monotherapy were well-tolerated. The atezolizumab was given intravenously every three weeks with a median OS of 10.1 months (95% CI: 7.3–17.0 months) and a 3-year OS rate of 27% (95% CI: 17–36%) [122]. A long-term follow-up study by van der Heijden et al. reported that atezolizumab has better efficacy than chemotherapy (vinflunine/paclitaxel/docetaxel) with an OS rate of 23% vs. 13% within 24 months [123]. Atezolizumab can also be an option as single-agent neoadjuvant immunotherapy for high-grade BCa treatment. In the ABACUS trial by Powles et al., 95 patients with MIBC treated with two cycles of atezolizumab before cystectomy had a pathological CRR of 31% (95% CI: 21–41%) [124]. According to the result of the updated safety analysis of the ABACUS trial reported by Szabados et al., the common adverse effects were fatigue (20%), decreased appetite (6%), and transaminitis (6%) [125]. A phase 1 trial study by Marcq et al. suggests that considerable caution with atezolizumab for MIBC should be considered [126].

Avelumab, durvalumab (anti-PD-L1), and nivolumab (anti-PD-1) are approved as alternative drugs for advanced or metastatic BCa. In the results of a phase 3 trial by Powles et al., OS at 1 year was 71.3% in the avelumab group and was 58.4% in the control group (median OS: 21.4 months vs. 14.3 months; HR: 0.69; 95% CI: 0.56–0.86) [127]. In a phase 2/3 multicenter study, patients with inoperable or metastatic solid tumors were treated with durvalumab, the objective response rate (ORR) was 31.0% (95% CI: 17.6–47.1%) in 42 response-evaluable patients, and 46.4% (95% CI: 27.5–66.1%) in the subgroup of PD-L1 was positive [128]. A good objective response was observed in 19 of 78 patients (24.4%, 95% CI: 15.3–35.4%) given nivolumab monotherapy in a multi-center phase 1/2 trial [129].

#### 4.1.2. CTLA-4 ICIs

CTLA-4, an inhibitory surface receptor expressed on activated Tregs, is the first FDA-approved ICIs for cancer treatment. It interferes with the binding of co-stimulatory molecules (CD80 and CD86) expressed on antigen-presenting cells (APC) to T cell surface receptors (CD28). Blocking the interaction between CTLA-4 and its ligand facilitates T cells to recognize and kill cancer cells, and does not deplete FOXP3+ cells in human tumors [130,131,132]. Three anti-CTLA-4 antibodies have shown promising results in cancer treatment, including ipilimumab, tremelimumab, and MK1308 [133]. In the CheckMate 032 trial, patients with platinum-pretreated metastatic disease were treated with nivolumab alone or combination therapy of nivolumab and ipilimumab. The results showed that the median duration of response was more than 22 months in all arms [134]. In addition, favorable efficacy and a well-tolerated profile of tremelimumab were demonstrated in a multi-center study (NCT02527434) [135].

M7824 is a bidirectional fusion protein, integrating both ICIs and TGF-β inhibition and regulating immune suppression in the tumor microenvironment. According to a phase 1 trial by Strauss et al., M7824 has a manageable safety profile for patients with advanced solid tumors [136].

### 4.2. 4-1BB Antibodies

4-1BB (CD137, tumor necrosis factor receptor superfamily 9) is an inducible co-stimulatory receptor expressed on activated T cells and natural killer (NK) cells. A 4-1BB connection on T cells triggers a signaling cascade that leads to the up-regulation of anti-apoptotic molecules and cytokines, and enhances effector function. In NK cells, 4-1BB signaling increases antibody-dependent cell-mediated cytotoxicity. The excitatory monoclonal antibodies targeting 4-1BB have been developed for cancer immunotherapy through the 4-1BB signaling pathway. The clinical trials of two agonist antibodies, urelumab and utomilumab, are ongoing [137]. The complete or partial responses were confirmed in 6 out of 23 patients (26.1%) with solid tumors in a phase 1b study (NCT02179918). The pharmacokinetics and immunogenicity of monotherapy and combinations of utomilumab and pembrolizumab were similar [138]. Urelumab has higher efficacy than utomilumab, but its clinical utility compromised inflammatory hepatotoxicity [137]. Further studies on investigating 4-1BB agonists for BCa patients are expected.

### 4.3. Interleukins

Interleukins are used in BCa treatment to enhance the immune response [107]. ALT-803 is a novel interleukin-15 (IL-15) superagonist complex to regulate lymphocytes and kill cancer cells. Compared with intravesical BCG alone, subcutaneous ALT-803 treatment or combined with BCG were well tolerated in the orthotopic BCa mouse model [139]. The first human trial also showed promising results [140,141].

N-803 (IL-15RαFc), an IL-15 analogue, is a recombinant protein fusion. In a phase 1 trial, eight of nine participants (89%) experienced complete response or progression within six months and eight (88.9%) were disease-free after 6-year treatment. The mean follow-up was 65.2 months (5.4 years), and only one patient had a recurrence after 38 months [142]. The phase 2/3 clinical trial of N-803 is ongoing.

NKTR-214 (bempegaldesleukinin) is a novel IL-2 pathway agonist that provides continuous signaling through the heterodimeric IL-2 receptor β/γ pathway and stimulating the proliferation and activation of CD8+ T cells [143,144]. The PIVOT-02 study is a phase 1 trial (NCT02983045) that NKTR-214 combined with either nivolumab or ipilimumab/nivolumab for immunotherapy-naïve advanced solid tumors. The total ORR was 59.5% (22/37) with seven complete responses (18.9%), which may meet the urgent need for new therapies in patients whose tumors lack PD-L1 expression [145].

### 4.4. Vaccine Therapy

MTBVAC is a live attenuated vaccine, derived from mycobacterium tuberculosis, which induces cell growth inhibition following internalization [146]. The results of an in vivo test in the orthotopic murine model of BCa showed that MTBVAC had better antitumor activity than BCG [146]. Tameris et al. conducted an incremental drug trial, which indicated the acceptable responsiveness of MTBVAC treatment [147].

VPM1002BC is a modified BCG vaccine for NMIBC with BCG failures. Rentsch et al. showed that intravesical instillation with VPM1002BC was well-tolerated, resulting in a potential Th1-weighted immune response [148].

PANVAC is a poxvirus vector-based vaccine derived from two viral vectors (recombinant bovine pox and chicken pox) [149,150]. The results of a phase 2 study of the combination therapy of PANVAC with BCG showed no significant difference between the combination group and BCG alone in RFS within 12 months [151].

BN-CV301 is another poxvirus-based vaccine containing encoded tumor-associated antigens and co-stimulatory molecules (MUC1, CEA, B7.1, ICAM-1, and LFA-3), which shows more powerfully antigenic properties than PANVAC.

Vesigenurtacel-L (HS-410), a whole-cell allogeneic vaccine, had confirmed anti-tumor activity for NMIBC [152]. Overall, there have been many recent reports on vaccine therapy, which is a promising method for future tumor treatment.

### 4.5. Oncolytic Viruses

Oncolytic viruses are natural viruses replicating within tumor cells to kill them selectively [153]. Coxsackievirus A21 (CVA21), a novel intercellular adhesion molecule-1 (ICAM-1) virus, was applied for tumor-targeted immunotherapy. Annels et al. enhanced the replication and oncolysis of CVA21 by increasing the surface expression level of ICAM-1. They clarified that ICAM-1 and plasmacytoid dendritic cells were critical factors for the remarkable therapeutic effects in the MB49 BCa model [154,155]. The phase 1 trial demonstrated that either ICAM-1 alone or combined with MMC exhibited a good safety profile in NMIBC patients [156].

CG0070, a conditionally replicating GM-CSF expressed on oncolytic adenovirus, showed that the CRR for patients with NMIBC was 63.6–81.8% [153,157,158]. In a phase 2 study by Packiam et al., patients with BCG-unresponsive NMIBC treated with intravesical CG0070, the results showed an overall CRR was 47% (95% CI: 32–62%) within six months [159].

Nadofaragene firadenovec (rAd-IFNa/Syn3), a replication-deficient recombinant adenovirus, introduced human IFN-α -2b cDNA into the bladder epithelium for BCG non-responsive NMIBC, and 55 of 103 patients (53.4%) with CIS achieved complete response within three months after the first instillation [160].

**Table 5:** The results of immunotherapies and targeted therapies.

Types	Agents	OS/ Median OS	HR	CI	CRR	ORR	References
anti-PD-1 ICI	pembrolizumab	10.3 m (14.1 m)	0.73	0.59–0.91%			[113]
anti-PD-1 ICI	pembrolizumab				0.4		[115]
anti-PD-L1 ICI	atezolizumab	10.1 m		7.03–17.0 m			[122]
		27%		17.0–36.0%			
anti-PD-L1 ICI	avelumab	71.3% (1y)	0.69	0.56–0.86			[127]
anti-PD-L1 ICI	durvalumab			17.6–47.1%		31.0%	[128]
anti-CTLA-4 ICI	nivolumab/nivolumab + ipilimumab					25.6%/26.9%	[134]
anti-CTLA-4 ICI	tremelimumab			7.2–36.4	6.3%	18.8%	[135]
4-1BB antibodies	urelumab + utomilumab				26.1%		[138]
IL-15 analogue	N-803	65.2 m (5.4y)					[142]
IL-2 agonist	NKTR-214				18.9%	59.5%	[145]
oncolytic viruses	CG0070				63.6–81.8%		[153,157,158]
				32%–62%	47% (6 m)		[159]
oncolytic viruses	nadofaragene firadenovec				53.4% (3 m)		[160]
FGFR inhibitors	erdafitinib				3.0%	40.0%	[161]
					30–49%	40.0%	[162]
anti-VEGF + GC	bevacizumab	14.3–14.5 m (76.3 m)	0.87	0.72–1.05			[105]
anti-VEGF	ramucirumab			18.8–30.3%		24.5%	[163]
antibody-drug conjugates	enfortumab vedotin			35.1–53.2%		44.0% (10.2 m)	[164]
		12.88	0.7	0.56–0.89			[165]
antibody-drug conjugates +	enfortumab vedotin + pembrolizumab				14.0%	62.01%	[166]
antibody-drug conjugates	sacituzumab govitecan	5.4 m		3.5–7.2 m			[167]
		10.9 m		9.0–13.8 m			

OS = overall survival; HR = hazard ratio; CI = confidence interval; CRR = complete response rate; ORR = objective response rate; ICI = immune checkpoint inhibitor; m = month; y = year; IL-15 = interleukin-15; GC = gemcitabine + cisplatin.

## 5. Targeted Therapy

### 5.1. Tyrosine-Kinase Inhibitors

#### 5.1.1. FGFR Inhibitors

Fibroblast growth factor receptor (FGFR) is a tyrosine kinase involved in the survival and the proliferation of tumor cells. FGFR is involved in proliferation, differentiation, migration, and survival of cells. When FGFR is overexpressed, the FGFR signaling pathway is activated, leading to normal cell carcinogenesis through the RAS-RAF-MAPK, STAT, PLCγ, and PI3K-AKT signaling pathways [168,169].

Erdafitinib, a pan-FGFR inhibitor, is the first FDA-approved FGFR inhibitor to treat advanced BCa. In a phase 2 study by Loriot et al., erdafitinib was used to treat high-grade/metastatic BCa with FGFR alterations after chemotherapy or neoadjuvant therapy, and the ORR was nearly 40% (CRR of 3% and partial response rate of 37%) [161]. A recent long-term follow-up study by Radtke et al. also confirmed the therapeutic activity of erdafitinib with an ORR of 40% (95% CI: 30–49%) and adverse events of stomatitis and hyponatremia of 14% and 11%, respectively [162].

Infigratinib (BGJ398) is another oral pan-FGFR kinase inhibitor. Nogova et al. proved its antitumor activity (seven patients with partial responses) for patients with FGFR2 fusions in a phase 1 dose-escalation and dose-expansion study [170]. Besides, several studies reported that the combination of anti-PD-1 and dasatinib (a tyrosine kinase inhibitor of DDR2) reduced tumor load in non-small cell lung cancer mouse model [171,172].

Other potential FGFR inhibitors have shown the positive effect for cancer treatment, including rogaratinib [173], pemigatinib [174], derazantinib (ARQ 087) [175], Debio 1347 [176], futibatinib [177], and vofatamab [178]. Combination strategies involving FGFR inhibitors with other agents may enhance the therapeutic effect or prevent drug resistance.

#### 5.1.2. HER2-Targeted Agents

The human epidermal growth factor receptor (HER) family is a tyrosine kinase receptor consisting of EGFR, HER2, ErbB3, and ErbB4, which is associated with the formation and progression of malignant tumors. The over-expression and/or amplification or other mutations of HER2 are associated with the recurrence and metastasis of many solid tumors, including biliary tract, colorectal, non-small-cell lung, and bladder cancers. EGFR amplification (11%), HER2 amplification (7%), and somatic ErbB3 mutations (11%) were more common in BCa [179,180]. HER2-targeted therapies have been primarily treated for breast cancer, and there are few studies on BCa management. Several HER2-targeted agents have been discovered, including antibodies (trastuzumab, pertuzumab), antibody–drug conjugates (trastuzumab emtansine), and small-molecule kinase inhibitors (lapatinib) [181]. Among of them, lapatinib may have a dual inhibitory effect in BCa patients with HER2/EGFR over-expression, indicating that patients with low expression of HER2/EGFR would prolong survival by lapatinib treatment [179,181].

Afatinib has shown significant activity for platinum-refractory metastatic BCa with HER2 or ErbB3 alterations, and may serve as a potential target for enhancing the efficacy of radiotherapy in unresponsive tumor cells [179,182]. Maeda et al. used lapatinib to treat dogs with urothelial carcinoma, reporting that dogs treated with lapatinib and piroxicam had smaller primary tumor sizes and higher survival rates than that of those treated with piroxicam alone [183].

Jack et al. developed a new therapeutic strategy that combines EGF with an anthrax toxin proposed for BCa, and the toxin is specifically taken up by cancer cells and induces rapid apoptosis after intravesical application, regardless of whether the cancer cells express Her2. About 30% of average tumor reduction after one treatment cycle was found in dogs with spontaneous BCa, who had failed or were not eligible for other therapies [184].

### 5.2. Anti-Angiogenesis

Angiogenesis plays a key role in the developing of many malignancies, especially urothelial carcinoma. Numbers of angiogenesis biomarkers are associated with the prognosis of metastatic diseases. The use of bevacizumab (anti-VEGF: anti-vascular endothelial growth factor) offers a new promising treatment for patients who are undergoing cisplatin-based chemotherapy and neoadjuvant immunotherapy [179,185]. In a randomized phase 3 study (CALGB 90601), gemcitabine and cisplatin (GC) were combined with bevacizumab or placebo and were used for patients with advanced urothelial carcinoma. The median follow-up time of surviving patients was 76.3 months, with the median OS of 14.3–14.5 months (HR: 0.87; 95% CI: 0.72–1.05).

Ramucirumab, a monoclonal antibody targeting VEGFR-2, has been tested in various tumors [186]. Petrylak et al. conducted a phase 3 trial that 530 patients were randomized to ramucirumab plus docetaxel (*n* = 263) or placebo plus docetaxel (*n* = 267) groups. The results showed the ORR was 24.5% (95% CI: 18.8–30.3%) and 14.0% (95% CI: 9.4 -18.6), and the ramucirumab group had 20% of serious adverse events [163]. However, most of the VEGF-targeted agents provided disappointing results when used alone, or led to unacceptably high toxicity when used in combination with other drugs.

### 5.3. Antibody–Drug Conjugates

Antibody–drug conjugates (ADCs) are immune conjugates linked with monoclonal antibodies and cytotoxic drugs via chemical connectors [187,188,189], which delivers cytotoxic loads specifically to the target cells, resulting in the death of targeting cells. Besides, adjacent tumor cells and surrounding stromal tissues are also attacked by activating the complement system and triggering immune effector cells of the tumor site [187]. It has been reported that nectin-4 and trop-2 would increase their expression in the bladder after the BCG strategy [190,191].

Enfortumab vedotin, an ADCs targeting nectin-4, was approved by the FDA for the patients who progressed following chemotherapy and immunotherapy. In a phase 2 study (EV-201), patients with advanced BCa received platinum-chemotherapy and anti-PD-1/L1 therapy were treated intravenously with enfortumab vedotin. The median follow-up was 10.2 months (range 0.5–16.5 months), and the confirmed ORR was 44% (95% CI: 35.1–53.2%). The most frequent adverse events were fatigue, peripheral neuropathy, alopecia, rash, decreased appetite, and dysgeusia [164]. In a recent phase 3 trial by Powles et al., the OS was longer in the enfortumab vedotin group than in the chemotherapy group (median OS: 12.88 vs. 8.97 months; HR: 0.70; 95% CI: 0.56–0.89; *p* = 0.001) [165]. According to Hoimes et al., patients with metastatic BCa were treated with enfortumab vedotin plus pembrolizumab with an ORR of 62% and a CRR of 14% [166]. A recent safety evaluation of enfortumab vedotin from McGregor et al. showed no deterioration in the quality of life and functional capacity were found in patients with advanced BCa [192].

Sacituzumab govitecan is a TroP-2-directed ADCs. According to a phase 2 study by Tagawa et al., a positive result of sacituzumab govitecan for patients with locally advanced MIBC was obtained. The median duration of response was 7.2 months (95% CI: 4.7–8.6), and the median PFS and OS were 5.4 months (95% CI: 3.5–7.2) and 10.9 months (95% CI: 9.0–13.8), respectively [167].

## 6. Novel Therapy

### 6.1. Novel Drug Delivery System

The multiple cellular layers of the urinary epithelium form a barrier within the bladder to pathogens, urine, and its associated metabolites. To enhance the efficacy of intravesical instillation, researchers developed physical, chemical, and carrier delivery strategies to improve the permeability of intravesical agents [7,193,194,195,196]. Hydrogel has become a hotspot in intravesical drug delivery systems in recent years. TC-3, a hydrogel system, could control drug release and adhere to bladder epithelium, with minimal adverse reactions and good safety [197].

UGN-101 (Jelmyto), a new formulation of MMC and chitosan, was designed to increase urinary retention time and the therapeutic efficacy of intravesical pharmaceutics. Preliminary results from a phase 3 clinical trial (OLYMPUS study) exhibited that the UGN-101 was a successful chemoablative agent for treating low-grade BCa, and its safety was confirmed in a study by Matin et al. [198,199]. The well-tolerated properties of UGN-102 as a mitomycin-containing reverse thermal gel (Optima II) were demonstrated in a phase 2b study by Chevli et al. [200]. Moreover, chitosan could be developed into different forms, such as thin films, nanoparticles, sponges, and hydrogels. Chitosan-based hydrogels can be used to deliver various drugs, such as antibiotics, anesthetics, or anticancer drugs [201]. According to a study from Zhang et al., a magnetic thermosensitive hydrogel for intravesical BCG was developed, which significantly prolonged the residence time of BCG in the bladder under an external magnetic field. Compared with conventional BCG treatment for NMIBC, the gel-based BCG system induced a more robust Th1 immune response and showed a higher anti-tumor efficacy [202].

Overall, the research on drug delivery carriers and nanoparticles and the pursuit of enhancing drug efficacy does not stop here. Materials including chitosan, liposomes, and polymeric materials have also been promising carriers for novel drug delivery in recent years. More comprehensive clinical trials are needed in the future.

### 6.2. Photodynamic Therapy

Similar to PDD, photodynamic therapy (PDT) induces cell death through the production of reactive oxygen species [203,204]. Filonenko et al. reported that PDT with 5-ALA caused 22% recurrence for patients with NMIBC in the first year after treatment [205]. Moreover, some metal compounds, such as purlytin, lutrin/antrin, photosens, TOOKAD soluble, and TLD1433, can be alternative photosensitizer options. TLD1433, a Ru (II) polypyridyl complexes, is a novel photosensitizer for PDT with photochemical and photophysical properties to provide energy and electron transfer, resulting in oxygen-dependent and/or oxygen-free photobiological activity. The effectiveness and safety of TLD1433 for NMIBC treatment were confirmed in a phase 1 trial [206,207]. Kustov et al. used TURBT and a combination of fluorescence diagnosis and PDT with chlorin e_6_-typed photosensitizers to treat patients with NMIBC. A median follow-up of 24 months (range 16–35 months) and 11 of 12 patients with tumor-free were obtained [208]. Overall, PDT is gradually incorporated into the management of BCa as a non-invasive therapy.

## 7. Systemic Therapy

### 7.1. Neoadjuvant Chemotherapy

For patients who are unsuitable/unwilling to undergo RC, trimodal therapy (TMT) with maximal TURBT and concurrent chemoradiation are potential alternatives to RC for MIBC [2,209,210,211]. TMT is an alternative regimen to RC, in which patients are treated with maximal TURBT followed by radiosensitizing chemotherapy and radiation. The use of chemotherapy in TMT is common, and regimens could be a combination of cisplatin and fluorouracil/paclitaxel, fluorouracil with MMC, or cisplatin-alone, and low-dose gemcitabine (depending on the grade of BCa) [2,212]. According to a study from Royce et al., the lifetime outcomes are evaluated after TMT and RC with/without neoadjuvant chemotherapy for 67-year-old patients with MIBC. TMT was the most effective treatment, with an incremental gain of 0.59 quality-adjusted life years (QALYs) over RC (7.83 vs. 7.24 QALYs, respectively) [212].

Clinical practice guidelines in oncology include three recommended neoadjuvant chemotherapy regimens: cisplatin, methotrexate, and vinblastine (CMV); methotrexate, dose-dense vinblastine, doxorubicin, and cisplatin (ddMVAC); and gemcitabine and cisplatin/carboplatin (GC) [213,214]. For metastatic BCa, the GC regimen showed better and significantly lower toxicity than the ddMVAC regimen, which was better-tolerated [210,211,215]. The efficacy of ddMVAC was validated in a phase 2 prospective study by Lyer et al., with 57% of patients meeting the endpoint (<pT2N0) [216]. Pfister et al. showed that significantly higher 3-year PFS in the ddMVAC group relative to the GC group (66% v 56%, HR: 0.70; 95% CI: 0.51–0.96, *p* = 0.025) [217]. Another study of a triple-drug regimen of MMC, doxorubicin, and cisplatin (MDP) for patients with newly diagnosed papillary NMIBC reported that DFS is similar to those treated with BCG. The MDP group had fewer side effects leading to discontinuation (5.8% vs. 15%) [104].

### 7.2. Neoadjuvant Combination of Immunotherapy and Chemotherapy

Bladder-sparing patients may benefit from the combination of immunotherapy and neoadjuvant therapy. Funt et al. reported that the primary endpoint (< pT2N0) was met in 27 of 39 (69%) patients with MIBC who were treated with neoadjuvant atezolizumab and GC in phase 2 trial, containing 16 (41%) pT0N0 [218]. Neoadjuvant therapy combined gemcitabine with split-dose cisplatin plus pembrolizumab for patients with MIBC also showed positive results that 22 of 39 patients (56%, 95% CI: 40–72) achieved a primary endpoint [219]. Petrylak et al. reported the efficacy and safety of docetaxel in combination with ramucirumab (human IgG1 VEGFR-2 antagonist) or placebo for the platinum-refractory MIBC treatment. The result showed that overall OS was prolonged significantly in the ramucirumab group than that in the placebo group (9.4 months, 95% CI: 7.9–11.4 vs. 7.9 months, 95% CI: 7.0–9.3) [220]. Moreover, Crabb et al. found that guadecitabine (DNA methyltransferase inhibitor) combined with GC for metastatic solid cancer treatment had a well-tolerated profile [221].

### 7.3. Novel Surgical Approach

With the innovation of surgical approaches, laparoscopic minimally invasive surgery has gradually replaced traditional open surgery. Surgery through minimally invasive techniques causes faster recovery by reducing intestinal manipulation, fluid displacement, and limiting the patient’s unconscious loss, thereby reducing gastrointestinal, nutritional, and cardiovascular morbidity [222]. In recent years, robot-assisted surgery has been gradually incorporated into the treatment for locally advanced BCa. Retrospective data indicated that the positive margin rate and lymph node quality of robot-assisted RC were similar to large, multi-institution, open RC operations [223].

## 8. Conclusions

With the rapid development of disciplines and technologies, clinical management protocols are constantly being revolutionized. The diagnostic techniques for BCa gradually become non-invasive with the aim of decreasing the risk of infection and injury and maintaining highly precise diagnosis. The incorporation of optical techniques and imaging tools significantly improved the options and precision of BCa diagnosis. With the development of interdisciplines, multidisciplinary cooperation is widely employed in medical research, especially in oncology. Deep learning is a great example, which is involved in diagnostic approaches, providing intelligent systems to facilitate the diagnostic manner. For non-invasive urine tests, the types of biomarkers have expanded from proteins to extracellular vesicles, meeting the stringent requirements for effective monitoring of BCa.

On the other hand, the treatment of MIBC and metastatic disease have expanded to immunotherapy and targeted therapies. Applying ICIs in neoadjuvant therapy offers promising options for MIBC patients who are ineligible for cisplatin-based chemotherapy. Although the treatment options for BCa emerged are endless, the willingness and needs of patients may not be achieved in different cases. The utility of biomarkers and gene sequencing may play a beneficial role. Moreover, multidrug combination strategies have gradually become the mainstream therapy for BCa. Finally, strategies including novel drug delivery systems, non-invasive PDT method and novel surgical approaches enrich the BCa management.

Overall, the development of safe and convenient diagnostic technologies and therapeutic drugs are always the driving force of further research for precise diagnosis and treatment.

## Figures and Tables

**Table 1:** FDA-approved biomarkers and urine protein markers.

FDA-Approved Biomarkers	Markers	Method	Sensitivity/%(95% CI)	Specificity/%(95% CI)
NMP22 BC	Nuclear mitotic apparatus protein	ELISA	69 (62–75)	77 (70–83)
NMP22 BladderChek	Nuclear mitotic apparatus protein	Point-of-care test	58 (52–59)	88 (87–89)
BTA Stat	Complement factor H-related protein	ELISA	65 (57–82)	74 (68–93)
BTA TRAK	Complement factor H-related protein	Point-of-care test	64 (66–77)	77 (5–75)
UroVysion	Alt in chromosomes 3, 7, 17, and 9p21	FISH	72 (69–87)	83 (89–96)
uCyt+/ImmunoCyt	Carcinoembryonic antigen, bladder tumor cell-associated mucins	Fluorescent immunohistoche- mistry	73 (68–77)	66 (63–69)

FDA = Food and Drug Administration; NMP = Nuclear matrix protein; UBC = Urinary Bladder Cancer; MCM5 = Microchromosome maintenance protein 5; ELISA = Enzyme-linked immunosorbent assay; FISH = Fluorescence in situ hybridization.

**Table 2:** Urinary biomarkers assays for BCa.

Urinary Biomarker Tests/Biomarkers	Markers	Method	Sensitivity/ %(95% CI)	Specificity/ %(95% CI)
CYFRA21-1	Cytokeratin 19 (cytoskeletal protein)	ELISA	82 (0.70–0.90)	87 (0.84–0.90)
UBC	Cytokeratin 8 and 18 (cytoskeletal proteins)	ELISA	59 (0.55–0.62)	76 (0.72–0.80)
Survivin	A member of inhibitors of apoptosis gene family	Bio-dot test	79 (0.73–0.84)	87 (0.79–0.92)
BLCA-1	Nuclear matrix protein	ELISA	80	87
BLCA-4	Nuclear matrix protein	ELISA	93 (0.90–0.95)	97 (0.95–0.98)
ADXBLADDER	Microchromosome maintenance protein 5(MCM5)	ELISA	44.9 (36.1–54)	71.1 (68.5–73.5)
URO17	Keratin 17(cytoskeletal proteins )	Immunocytoche- mistry	100	96
Microsatellite analysis	DNA mutation	PCR	58–92	73–100
TRAP	Telomerase		90	88
Quanticyt	Cell nucleus	quantitative	59	79
HA-HAase			91	70
EIF5A2, AIB1 and NMP22 model			92	92
Cxbladder	mRNAs (IGFBP5, HOHA13, MDK, CDK1)		91 (0.85–0.95)	61 (0.21–0.90)
Xpert bladder cancer			72 (0.63–0.80)	76 (0.72–0.81)
Uromonitor			93 (0.79–0.98)	79 (0.62–0.90)
Oncuria™			93	93

ELISA = enzyme-linked immunosorbent assay; FISH = fluorescence in situ hybridization.

**Table 3:** DNA methylation assays and biomarkers for BCa detection.

Tests	Sensitivity (%)	Specificity (%)	PPV (%)	NPV (%)
Bladder EpiCheck	74 (95% CI: 57–85)	84 (95% CI: 80–88)	48 (95% CI: 42–54)	94 (95% CI: 90–97)
UroMark	98	97		97
utMeMA	90	83.1	>85	>85
Bladder CARE	93.5	92.6	87.8	96.2
The GynTect^®^	60	96.7		

PPV = positive predictive value; NPV = negative predictive value; CI = confidence interval.

**Table 4:** Non-exhaustive overview of urinary genetic biomarkers and extracellular vesicles biomarkers for BCa [48,82,83].

Genetic Biomarkers/Markers	Types	
TERT	DNA mutational analysis	
FGFR3	DNA mutational analysis	
Chromosomes	Microsatellite analysis	
CDK1, HOXA13, MDK, IGFBP5	Multigene panels	
Lactate, β-hydroxypyruvate, palmitoyl sphingomyelin, phosphocholine, arachidonate, BCAAs, adenosine, succinate	Metabolite biomarkers	
**Extracellular Vesicles Biomarkers**	**Types**	**Purposes**
Uroplakin-1	Transitional epithelial cells	Diagnosis
Uroplakin-2	Transitional epithelial cells	Diagnosis
TACSTD2	Protein	Diagnosis
EDIL-3	Protein	Diagnosis
Periostin	Protein	Prognosis
CD10, CD36, CD44, 5T4, CD147(basigin), CD73(NT5E), integrinβ1, integrinα6, Mucin-1(MUC1)	Protein	Diagnosis
Alpha-1-antitrypsin, histone H2B1K	Protein	Diagnosis
Resistin, GTPase NRas, EPS8L1, mucin 4, EPS8L2, retinoic acid-induced protein 3, ɑ subunit of GsGTP, binding protein, EH-domain-containing protein 4	Protein	Diagnosis
MAGEB4, NMP-22	mRNA, Protein	Diagnosis
FOLR1, TTP1	Protein	Diagnosis
TACSTD2	Protein	Diagnosis
miR-375, miR-146a	miRNA	Prognosis
miR-4454, miR-205-5p, miR-200c-3p, miR-200b-3p, miR-21-5p, miR-29b-3p, miR-720 /3007a	miRNA	Diagnosis
miR-200a-3p; miR-99a-5p; miR-141-3p; miR-205-5p	miRNA	Diagnosis
miR-15a-5p, miR-31-5p, miR-21, miR-155-5p, miR-132-3p	miRNA	Diagnosis
miR-940, miR-191, miR-93, miR-200c, miR-15a, miR-30a-3p, miR-503-5p, Mirlet7b	miRNA	Diagnosis
miR-66-3b	miRNA	Diagnosis
miR-146-5p, miR-138-5p, miR-144-5p	miRNA	Diagnosis
miR-145-5p, miR-23b	miRNA	Diagnosis
miR-133b	miRNA	Diagnosis
miR-375-3p	miRNA	Diagnosis
miR-29c	miRNA	Diagnosis
HOTAIR, HOX-AS-2, MALAT1 OCT4, SOX2	mRNA, lncRNA	Diagnosis
UCA1-201, UCA1-203, MALAT1, LINC00355	lncRNA	Diagnosis
SNHG16, Linc-UBC1		Diagnosis
PCAT-1		Diagnosis
H19		Diagnosis
LASS2, GALNT1, FOXO3, ARHGEF3	mRNA	Diagnosis
MDM2, ERBB2, CCND, CCNE1, CDKN2A, PTEN, RB1	DNA	Diagnosis

TERT = telomerase reverse transcriptase; FGFR3 = fibroblast growth factor receptor; miRNA = micro RNA; IncRNA = long noncoding RNA; TWIST1 = Twist-related protein 1, OSR1 = Protein odd-skipped-related 1, SIM2 = Single-minded homolog 2, OTX1 = Homeobox protein OTX1, MEIS1 = Homeobox protein Meis1, ONECUT2 = One cut domain family member 2.

## Abbreviation

Abbreviations are listed by the order of appearance in the manuscript.

Abbreviation	Meaning
BCa	bladder cancer
NMIBC	non-muscle-invasive bladder cancer
MIBC	muscle-invasive bladder cancer
FDA	food and drug administration
EVs	extracellular vesicles
TURBT	transurethral resection of bladder tumor
RC	radical cystectomy
WLC	white-light cystoscopy
CIS	carcinoma in situ
LIF	laser-induced fluorescence
AFC	autofluorescence cystoscopy
PDD	photodynamic diagnosis
5-ALA	5-aminolaevulinic acid
HAL	hexaminolevulinate
OR	odds ratio
CI	confidence interval
RFS	recurrence-free survival
NCV	numerical color value
OCT	optical coherence tomography
CLE	confocal laser endomicroscopy
AUC	the area under the receiver operating characteristic
NBI	narrow-band imaging
CT	computed tomography
MRI	magnetic resonance imaging
DWI	diffusion-weighted imaging
ADC	apparent diffusion coefficient
VI-RADS	vesical imaging-reporting and data system
DCE	dynamic contrast enhancement
mUS	micro-ultrasound
CEUS	contrast-enhanced ultrasonography
AI	artificial intelligence
CNN	convolutional neural networks
UBC	urinary bladder cancer
MCM5	microchromosome maintenance protein 5
NPV	negative predictive value
K17	keratin 17
TRAP	telomeric repeat amplification
FGFR 3	fibroblast growth factor receptor 3
HA-HAase	hyaluronic acid- hyaluronidase
EIF5A2	eukaryotic initiation factor 5A2
PPV	positive predictive value
GEO	gene expression omnibus database
TCGA	the cancer genome atlas
CFAU-II	cellular fluorescence analysis unit-II
MMC	mitomycin C
BCG	bacillus calmette-guérin
DFS	disease-free survival
PFS	progression-free survival
GCP	gemcitabine, cabazitaxel, and cisplatin
CRR	complete response rate
RFS	relapse-free survival
OS	overall survival
ICIs	immune checkpoint inhibitors
CTLA-4	cytotoxic T lymphocyte-associated protein 4
PD-1	programmed cell death-1
TGF-β	transforming growth factor-β
IDO	indoleamine 2, 3-dioxygenase
MDSCs	myeloid-derived suppressor cells
HR	hazard ratio
ORR	objective response rate
APC	antigen-presenting cells
NK	natural killer
IL-15	interleukin-15
FGFR	fibroblast growth factor receptor
HER	the human epidermal growth factor receptor
PDT	photodynamic therapy
TMT	trimodal therapy
QALYs	quality-adjusted life years
CMV	cisplatin, methotrexate, and vinblastine
ddMVAC	dose-dense vinblastine, doxorubicin, and cisplatin
GC	gemcitabine and cisplatin/carboplatin
MDP	MMC, doxorubicin, and cisplatin
